# The Book World of Medicine and Science

**Published:** 1900-11-10

**Authors:** 


### The Book World of Medicine and Science.

Thomas Sydenham. By Joseph Frank Payne, M.D., Oxon., Fellow and Harveian Librarian of the Royal College of Physicians. (Masters of Medicine Series.) London: T. Fisher Unwin. 1900. Price 3s. 6d.

With such a subject as Thomas Sydenham, the great practical physician who did so much by his works and precepts to give a bent to English medicine; with such a period for the setting of his tale as is given by the time of the civil war, of the plague, of the Restoration, and with the task entrusted to so careful a writer as Dr. Payne, it is not surprising that we have in this volume an interesting and suggestive record of a life and a pleasant literary exercise. Thomas Sydenham was born at Wynford Eagle and baptised September 10, 1624. He was the fifth son of William Sydenham, of Wynford Eagle and Mary his wife, daughter of Sir John Jeffery, Knt., of Catherston, and he was brought up in his country home under the influence of a conscientious father, a pious mother, and manly elder brothers.

At this time the nation was rent into parties, both in politics and in religion. It was in a strong Puritan and Parliamentary atmosphere that Sydenham passed his boyhood, and when he went to Oxford and entered at Magdalen Hall he found himself in what was the great centre of Oxford Puritanism. But Sydenham was not destined to profit very long by his association with his college, for his university career was cut short at its outset by political events. In the summer of 1642 the conflict between the King and the Parliament was rapidly proceeding to its final rupture. In May the Parliament called out the Militia in defiance of the King's prohibition; at the very moment when Sydenham was entering at Oxford, the trained bands in London were being drilled in Finsbury fields; and when in August the King raised his standard at Nottingham, the country was actually in a state of civil war. This gave the key to much of the drift of the first part of Sydenham's career. His family connections and the state of parties in his native county, placing him inevitably on the side of the Parliament, and Oxford siding with the King, Sydenham left the University, and instead of our finding him at eighteen devoted to serious studies, we see him at once involved in the exciting events of that period.

Five years later, namely, in 1647, Sydenham again went into residence in Oxford at his old college. Parliament was now triumphant, but although ordinances had been passed, the work of subjugating the University was still very imperfectly accomplished, and it is probable that Sydenham had but little opportunity for quiet study. He, however, entered as a Fellow Commoner of Wadham on October 14, 1647, and in accordance with the custom then frequently exercised, he was created Bachelor of Medicine by command of the acting Chancellor, the Earl of Pembroke, on April 14, 1648. Sydenham could not have then made any serious study of medicine, having been barely a year resident in the University which all the time was in a state of great confusion. Thus he became the possessor of a degree at the beginning instead of at the end of his student's course, and being a *persona grata* with the dispensers of patronage, soon found himself in comfortable circumstances; strange commentary upon Puritan rule.

Then we find him taking up military service once more, marrying, and settling in practice in King Street, Westminster, dabbling in politics, and even becoming a candidate for Parliament. Patronage again, after the manner of republics, rewarded political services, and Sydenham became "Comptroller of the Pipe." "Thus Patronage, with its customary blindness, having by mere luck made Sydenham a physician, was now about committing a disastrous blunder in making him an official. It is melancholy even to imagine that the great Sydenham might have passed his life sitting in an office signing leases." Providence, however, decided differently. Sydenham went to Montpellier where he probably studied under the celebrated physician Charles Barbeyrac, and then in 1661 we find him practising again in London under the new régime.

About the plague there is more perhaps in the book than the somewhat ignoble part played by Sydenham demands, for he seems to have followed his rich patients out of town until the epidemic had expended its greatest fury.

Dr. Payne gives a careful appreciation of the writings and the practice of Sydenham, and many details of his life, his friends, and his times, and altogether the story which he tells is an attractive and interesting presentment of the life of one who passed his time in the centre of the movement at a very interesting period of English history, as well as being a great leader in medicine.

Treatment by Hypnotism and Suggestion. By C. Lloyd Tuckey, M.D. Fourth Edition. Pp. 376. (London: Baillière, Tindall, and Cox. 1900. Price 7s. 6d.)

The last edition—published in 1880—of Dr. Tuckey's manual having been exhausted, the preparation of a new one has been made the occasion for revision and the incorporation of much new matter. In the last decade the position of hypnotism has materially changed. It cannot be said that very many medical men even yet have practical acquaintance with its phenomena. Yet the practitioner who employs hypnotism is no longer, we trust, regarded as a dabbler in immoral therapeusis, and much has been done to clear away the obscurities with which "false prophets" had surrounded the subject. Dr. Tuckey, as a follower of Liébault, to whom indeed he dedicates his book, regards hypnotism as a valuable therapeutic method. And his book is mainly, though not exclusively, devoted to a consideration of hypnotism as a means of cure. Although Dr. Tuckey has sat at the feet of Liébault, he is in no sense a prejudiced or unduly partial member of the School of Nancy, and while his great experience and much thought have often led him to take up positions antagonistic to those of the disciples at the Salpêtrière, yet is he willing to admit the natural connection between hypnosis and hysteria. Indeed, although Dr. Tuckey's statement of the theory of hypnosis scarcely leads us beyond the indubitable truth that the hypnotic state is subjective in origin and that its phenomena depend on "disconnection," he does much service in maintaining and illustrating the natural relationship of hypnosis and other nervous states, all probably dependent on "disconnection" or disassociation. Dr. Tuckey's work is, indeed, eminently sane, and he never allows his wholesome and justifiable belief in hypnosis to lead him into the vague transcendentalism of Myers and other authors. The records given by Dr. Tuckey of clinical cases are many and valuable: no exaggerated claims are made, and the most sceptical can hardly refuse them credence, whatever his opinion of the wisdom shown by the employment of hypnotism as a remedial measure.

